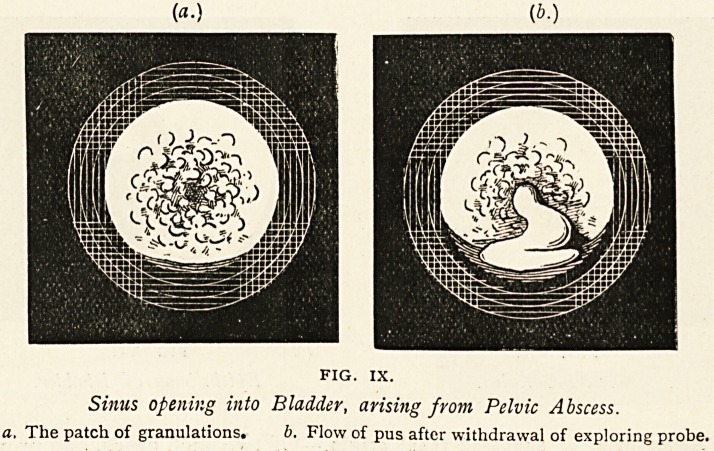# Cystoscopy and Ureteral Catheterization

**Published:** 1901-12

**Authors:** Thos. Carwardine

**Affiliations:** Assistant-Surgeon to the Bristol Royal Infirmary.


### CYSTOSCOPY AND URETERAL CATHETERIZATION.

### OBSERVATIONS ON TWENTY EXAMINATIONS FOR OBSCURE URINARY AND ABDOMINAL AFFECTIONS.

BY

THOS. CARWARDINE, M.S. (Lond.), F.R.C.S.,

*Assistant-Surgeon to the Bristol Royal Infirmary.*

DURING the last few years I have had several opportunities of testing the value of cystoscopy in both sexes, and of catheterization of the ureters in the female. The manipulations are often somewhat delicate and require considerable patience; but once the preliminary difficulties are surmounted, diagnoses may be arrived at which would be otherwise impossible. A reference to the table at the end of this article will show the frequent disparity between the provisional diagnosis and that arrived at by direct cystoscopic observation. I will classify the cases examined, and indicate their chief features.

**Tuberculous Kidney, diagnosed by Ureteral Catheterization.—Partial Nephrectomy.—Recovery.**

This was the case of a young woman who, having pulmonary tuberculosis also, was for some time treated for supposed tuberculous cystitis. She had pain on micturition, and a sensation as of something coming down at the time. The urine contained a considerable amount of albumin, and microscopically showed numerous pus cells and one or two red corpuscles in the field. Examination of the bladder with the cystoscopes showed it to be normal in every part, including the trigone. Catheterization of the ureters gave the following results:—

| R. | L. |
|---|---|
| Red corpuscles (few). | Red corpuscles (few). |
| Pyriform cells. | More debris. |
| Pus cells (very few). | Pus cells (numerous). |

Now in catheterization of the ureters slight bleeding is the rule. This accounted for the few red corpuscles present. In the specimen from the right side the few pus cells were probably contaminations. The prominent fact was the pus in the urine from the left ureter. To confirm this, catheterization of the left ureter was again performed later. The catheter was passed with ease for about six inches, and urine flowed at the rate of two drops per minute after the first gush of about a drachm into the test-tube. This urine was acid, deposited nearly double the amount of pus precipitated from the mixed urine, and Dr. J. O. Symes reported it to contain perfectly characteristic tubercle bacilli. A left tuberculous kidney was thus diagnosed, and when the left kidney was subsequently brought on to the loin the condition in Fig. I. was seen.

FIG. I.

*Tuberculous Kidney.*

*a.* Abscess. *t.* Tubercles.

At the top of the kidney was a large abscess, and towards the lower end there were several superficial miliary tubercles. A partial nephrectomy was performed, including the large abscess, and the patient recovered. Within fourteen days of the operation the albumin and pus had almost completely disappeared from the urine.

The case illustrates renal tuberculosis without affection of the bladder, and, but for the evidences of the disease in the lungs, the case would have been a particularly suitable one for nephrectomy. Her doctor tells me that she is now dying of consumption.

### Tuberculous Ulceration of the Bladder.

Two cases of this description will be referred to :—

**Case 1** is that of a young girl, with intermittent hæmaturia, who complained of pain in the upper part of both buttocks and in the left loin. Her urine was alkaline, and contained pus, blood, phosphates, and a few oxalates; so that renal calculus was the first diagnosis. But on examining the bladder with the cystoscope, one saw, as in Fig. II., numerous superficial ulcers. On one occasion a patch of whitish membrane, surrounded by a congested base, occupied the left ureteral region, and could be peeled off with forceps. The smaller ulcers were irregular, from the size of a split-pea to that of a threepenny-piece, pale round the edges, and having deep red granulations at the base. The left ureter was catheterized for six inches, and a ten minutes' specimen drawn off, comprising 7 c. c. of urine, with very few pus cells (probably from the bladder), but tubercle bacilli were not found. At a later period bacilli were found in the urine passed from the bladder naturally. Treatment to the bladder was adopted; there was marked improvement in the symptoms, and the improvement was confirmed by cystoscopic observation.

FIG. II.

*Tuberculous Ulceration of Bladder.*

I understand that the left kidney was subsequently explored by another surgeon and that no stone was found, thus confirming the cystoscopic observations. This case is an example of primary tuberculosis of the bladder.

**Case 2** was a young woman, who had had pain above the pubes for four months, with pyuria. Some superficial ulceration of the bladder was detected by cystoscopic examination under anæsthesia (Fig. III.). Local bladder treatment was adopted with marked benefit, and the patient gained 6 lbs. in weight in a week. The ureters were not catheterized;

FIG. III.

*Ulceration of Bladder and Orifice of Left Ureter.*

nor would such a procedure have been wise in the absence o other than bladder symptoms in this case.

### Cases with Symptoms Suggestive of Vesical Calculus.

**Tuberculous Tumour.**—The patient was a lad who, though seventeen years of age, was only developing into puberty. He complained of pain at times in the lower belly when passing water, towards the end of micturition. "The water stops suddenly," he said; "I double up, and later the water comes away again." The urine contained blood. He was given an anæsthetic, and the bladder was sounded, but no stone detected. The urethra was too small for the cystoscope to pass *per urethram*. The bladder was opened above the pubes and Leiter's cystoscope passed into the bladder to illuminate it. A polypoid tumour was then seen, acting like a ball and socket valve upon the internal orifice of the urethra. The tumour was removed, with relief of the symptoms.

FIG. IV.
*Suprapubic Cystoscopy.*
*s.* Suprapubic Aperture. *p.* Polyp.

It is of particular interest, for a tuberculous tumour of the bladder must be a very rare event. That it was tuberculous, containing tubercles, giant-cells and tubercle bacilli, was demonstrated by microscopical examination.

**Myelitis.**—This case was diagnosed as lumbago or (?) renal calculus. On examination with the cystoscope, numerous deep sacculi and fleshy fasciculi were observed, the former having the appearance of deeply-cupped hernial depressions. The patient had been ill for six months, with inability to hold his water and sharp pains in the left side, with attacks of cystitis. He had girdle-pains in the lower chest, Argyll-Robertson pupil, increased right knee-jerk, but no ataxy. The figure indicates the state of the bladder in such a case, and no calculus was to be seen. From the appearances it is probable that the patient was subject to attacks of retention.

FIG. V.
*Bladder in a Case of Myelitis.*

**Hypertrophied Prostate.**—The patient had been examined for stone by his doctor two years previously. Recently, for a few days, almost pure blood was drawn off from the bladder, but patient went forty-eight hours without any relief whatever. He had had increasing difficulty of micturition for three or four years. The stream would suddenly stop and then gradually return. The frequency was greater by day, and there was pain above the pubes. The cystoscope showed well-marked fasciculi and very deep recesses. The fasciculi were very distinct at the apex of the bladder. There was a collar-like enlargement of the prostate; and, as showing a new fact learned by cystoscopy, the effects of catheterization for some months had resulted in a rough shaggy condition of the posterior part of the prostatic urethra (*see* Fig. VI.).

FIG. VI.

*Bladder resulting from Hypertrophied Prostate.*

**Calculus Undetected by the Sound.**—The patient complained of something stopping his water, and inability to hold it; of the passage of blood, and of pain suggestive of stone. He was sounded for stone a year previously, and on the present occasion, without result. The cystoscope revealed a light feathery calculus, which sparkled brilliantly like hoarfrost in the rising sun—a picture to be seen, but only imperfectly depicted (Fig. VII.). The calculus was removed by suprapubic lithotomy.

**Epithelioma of Bladder.**—The symptoms in this case were supposed to be due to a vascular caruncle which the patient had. This was removed, but hæmaturia continued. The bladder was accordingly examined with Leiter's cystoscope, and an epithelioma was seen towards the right side (Fig. VIII.). Examined by Kelly's cystoscope, the ulcerating tumour was seen projected in relief.

FIG. VII.

*Vesical Calculus.*

FIG VIII.

*Epithelioma of Bladder.*

**Hæmaturia, of supposed renal origin.**—Of two such cases, one proved to be a large irregular hard calculus which had evaded detection by the sound, perhaps from its having been encysted. In the other case the cystoscope showed the bladder to be normal, and a tumour was to be felt in the right loin.

### Pyuria of Uncertain Origin.

Of this condition there were three cases, not included under previous headings.

In one the pyuria was very slight; the bladder was seen to be normal when viewed by the cystoscope, and the patient soon got well. In another case, the pyuria was accounted for by chronic cystitis. The patient was an elderly lady, who had been treated for cystitis; but, owing to the persistence of symptoms, I was asked to examine her bladder. The cystoscope showed general fasciculation and slight sacculation everywhere explaining the persistence of trouble, and special intra-vesical treatment was advised. The condition of the bladder is the more noteworthy because the patient was a spinster without any such obstruction as one usually associates with a sacculated bladder.

In this and other cases the cystoscope has indicated that sacculation is a much more common phenomenon of vesical disorder than one formerly supposed. It is a condition which is made more obvious in the moist, distended state of the bladder than when seen on the *post-mortem* table.

One of the most interesting of these cases was recently under my care.

She was supposed to have abscess of the left kidney, associated with old pelvic trouble. I examined the bladder, and detected a granulating surface towards the right side, which bled slightly when touched, at about the position of the *right* ureteral orifice. There

(*a.*) (*b.*)

FIG. IX.

*Sinus opening into Bladder, arising from Pelvic Abscess.*

*a.* The patch of granulations. *b.* Flow of pus after withdrawal of exploring probe.

was no sign of any pus. This surface was gently probed, and the instrument pressed in for a short distance at the centre. On withdrawal of the probe there appeared a white string of pus, which

| NO. | PATIENT. Sex. | Age. | DOCTOR. | PROVISIONAL DIAGNOSIS. | METHOD. | OBSERVATION. | REMARKS. |
|---|---|---|---|---|---|---|---|
| 1 | Female. | 19 | Dr. W. | Cystitis. Tuberculous kidney. | Leiter and Kelly (ureter). | Bladder normal. Pus in urine from left ureter. | |
| 2 | ,, | ,, | ,, | ,, | Leiter. | Foregoing confirmed. | |
| 3 | ,, | ,, | ,, | ,, | Kelly (ureter). | Pus (containing bacilli) in urine from left ureter. | Partial nephrectomy. |
| 4 | Male. | 18 | — | Vesical calculus. | Suprapubic cystoscopy. | Polypoid tumour blocking urethral orifice. | Tumour removed. Bacilli demonstrated. |
| 5 | Male. | 61 | Dr. L. | Vesical calculus. | Leiter. | Hypertrophied prostate, with sacculated bladder. | Catheter life. |
| 6 | Female. | 24 | Dr. L. | Cystitis. | Kelly. | Superficial ulceration. | Intravesical treatment. |
| 7 | Male. | 48 | — | Lumbago. ? Renal calculus. | Leiter. | Numerous deep sacculi and fasciculi. | Myelitis. |
| 8 | Male. | 19 | — | ? Calculus, thrice sounded. | Leiter. | Small ovoid calculus. | Suprapubic lithotomy. |
| 9 | Female. | 17 | — | Renal calculus. | Kelly. | Superficial ulceration of bladder. | |
| 10 | ,, | ,, | — | ,, | Kelly. | Ditto. Also whitish membrane around left ureter. | Local treatment to bladder (Bacilli found in urine). |
| 11 | ,, | ,, | — | ,, | Kelly (ureter). | Left ureter catheterized, and disease of left kidney excluded. | |
| 12 | Male. | 54 | Dr. W. | Hæmaturia, ? renal. | Leiter. | Field obscured by a dark object. | Perineal lithotomy. |
| 13 | Female. | 70 | - | Hæmaturia. Vascular caruncle. | Leiter. | Epithelioma of bladder. | |
| 14 | | ,, | — | ,, | Kelly. | Epithelioma (side view). | No operation. |
| 15 | Female. | 17 | — | as above. | Kelly. | Great improvement observed. | |
| 16 | Female. | — | Dr. C. | Abscess of kidney. | Kelly. | Bladder and ureteral regions normal. | Pyuria ceased. |
| 17 | Female. | 58 | Dr. F. | Slight pyuria. | Leiter. | Bladder sacculated and fasciculated. | Chronic cystitis. |
| 18 | Female. | 40 | Dr. T. | Abscess of left kidney. | Leiter. | Urine became cloudy, but abnormality to right detected. | |
| 19 | ,, | ,, | ,, | ,, | Kelly. | Sinus opening into the right of bladder from left side. | Extraperitoneal drainage. |
| 20 | Female. | 50 | Dr. G. | Renal calculus or tumour. | Leiter. | Bladder normal. | Kidney explored. Malignant tumour. |

poured out on to the surface of the blood collected in the Kelly's tube, like a fountain of viscid cream (Fig. IX.). A soft catheter was passed several inches into this aperture, and was found to have curved round behind the uterus towards the *left* broad ligament, where a hard inflammatory mass could be felt. A diagnosis of a sinus from the left broad ligament was made, opening into the *right* side of the bladder. The inflammatory zone was drained by the extra peritoneal abdominal route, and the patient soon began to improve, and left for convalescence in the country.

Cystoscopy in general, and catheterization of the ureters in particular, have certain difficulties and contra-indications; but the cases here related will serve to show how valuable the method of diagnosis becomes in certain obscure abdominal disorders associated with symptoms referable to the urinary tract. Catheterization of the ureters is also frequently employed to indicate the position of the ureters during the removal of uterine and ovarian tumours.

*Note.*—Since the appended table of twenty cases was compiled, the writer has made three further cystoscopic examinations. In one, there was no visible lesion to explain an intermittent hæmaturia. In the second, a small villous growth near the left ureteral orifice was unexpectedly discovered. This was removed by the suprapubic route. In the third, there was a sacculated bladder, whilst a tumour overhung the end of the cystoscope and proved on suprapubic exploration to be a large epithelioma.

## Figures and Tables

**FIG. I. f1:**
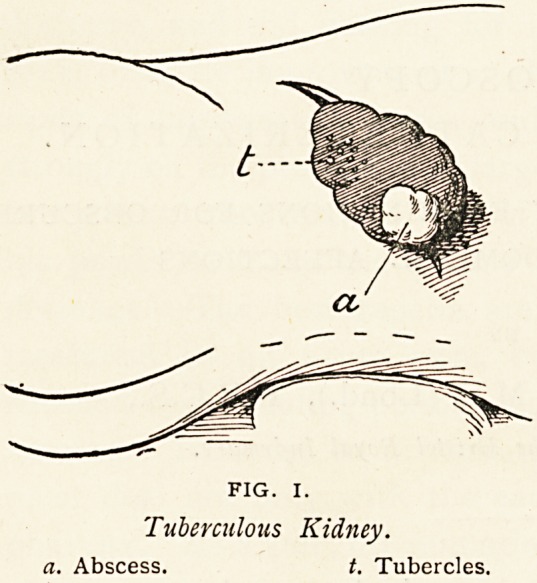


**FIG. II. f2:**
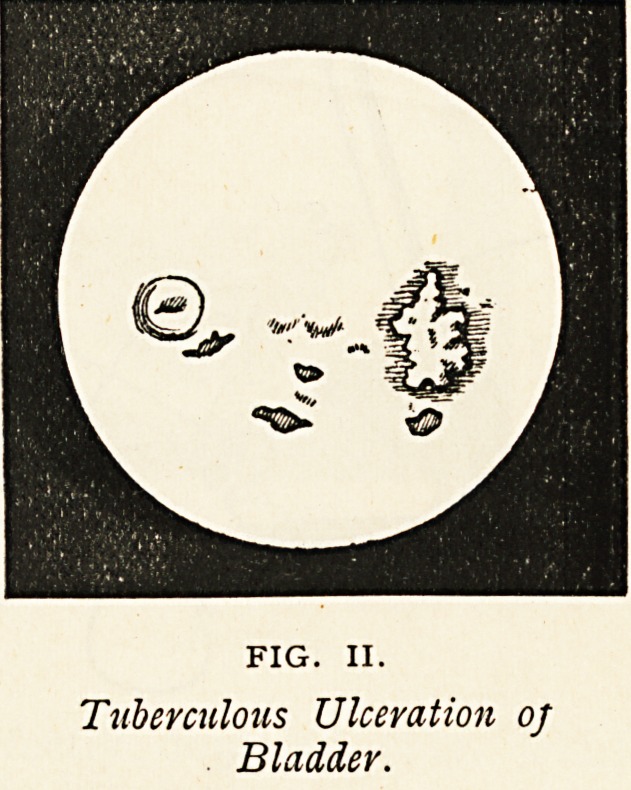


**FIG. III. f3:**
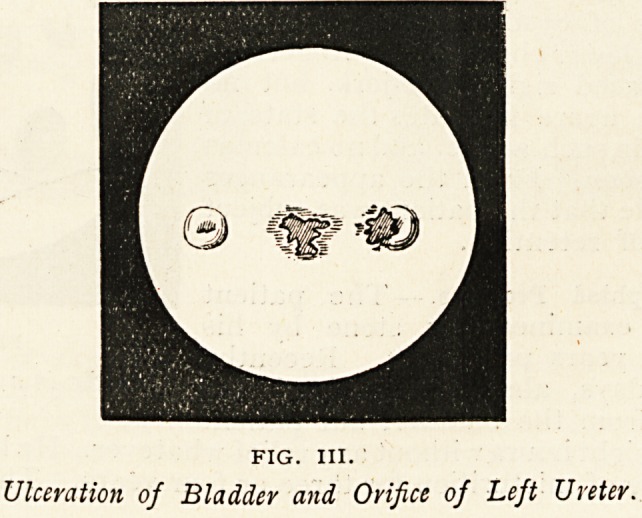


**FIG. IV. f4:**
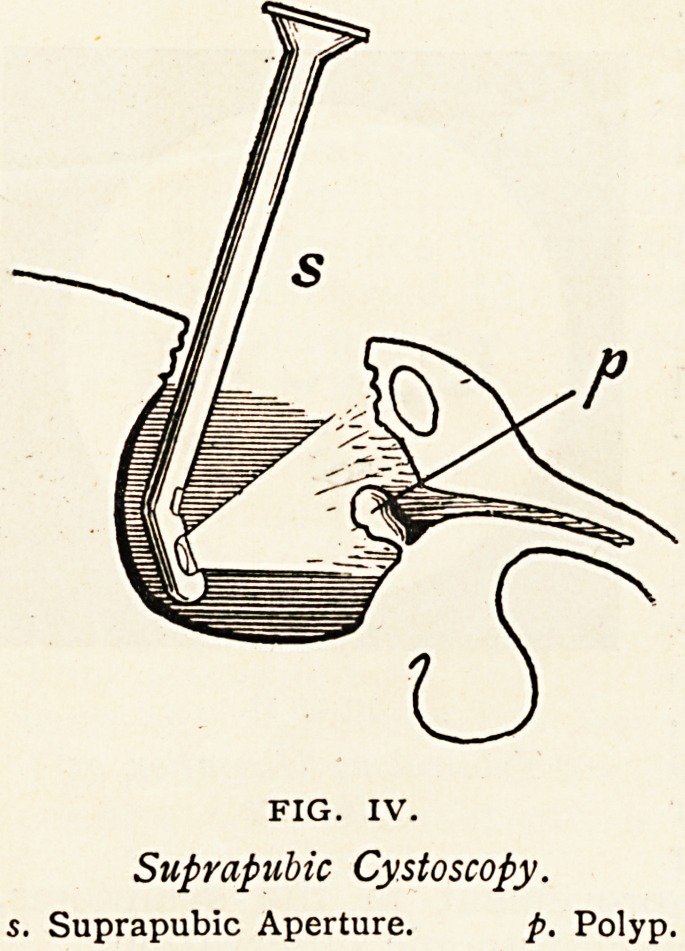


**FIG. V. f5:**
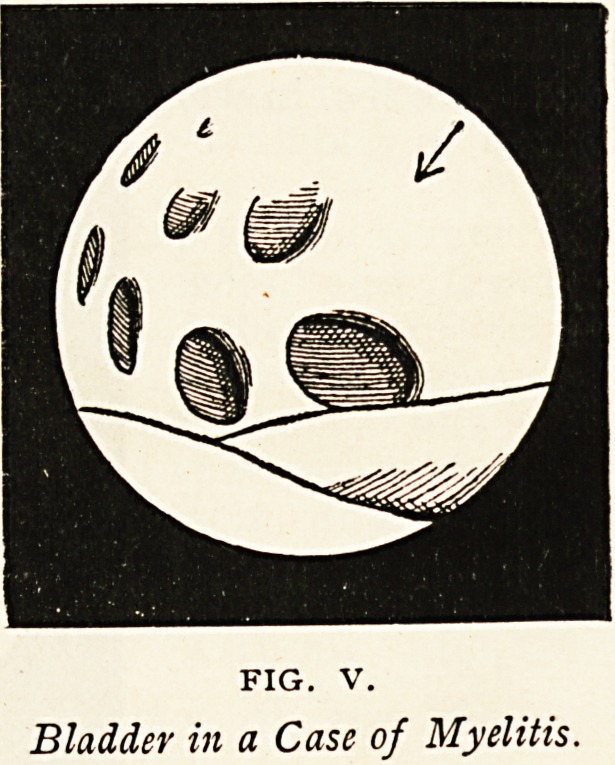


**FIG. VI. f6:**
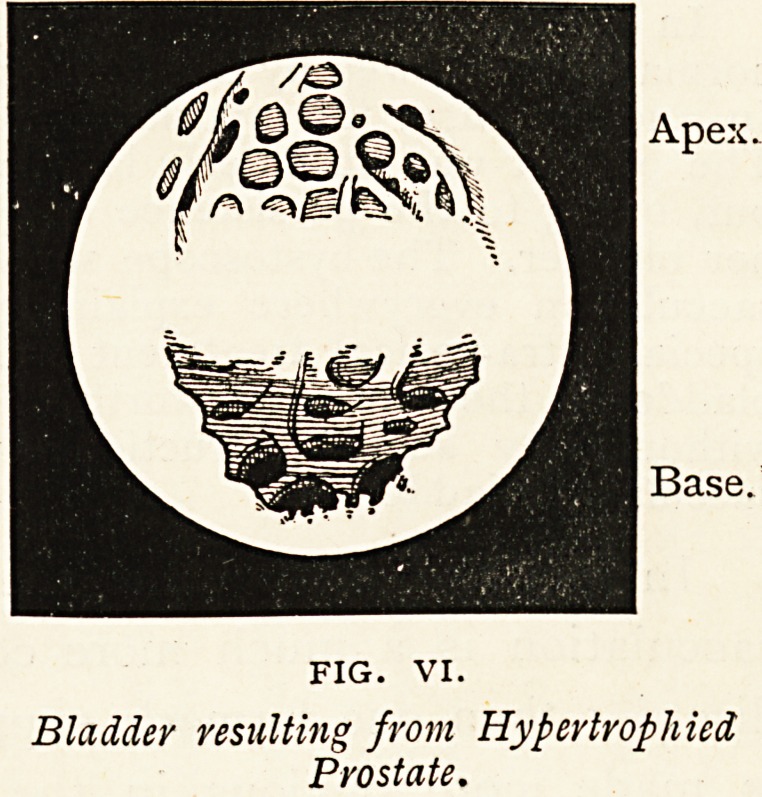


**FIG. VII. f7:**
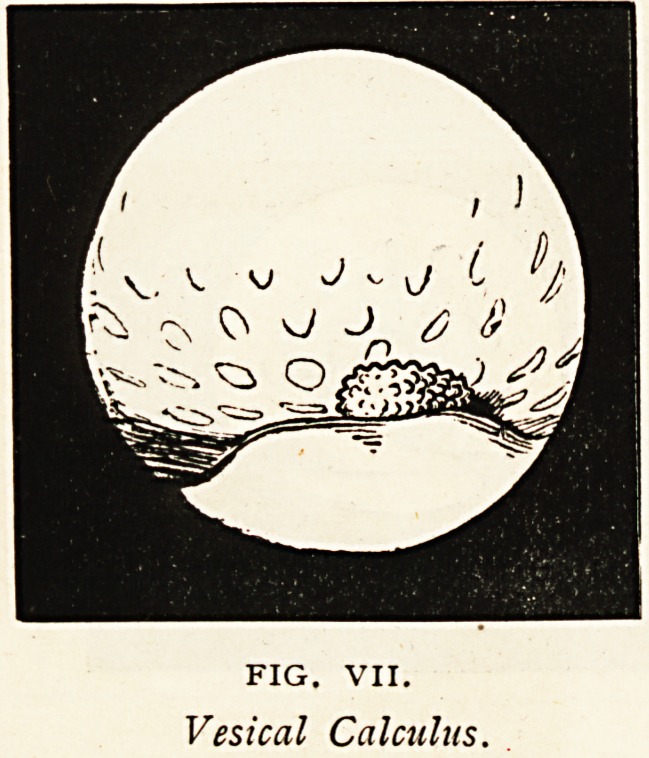


**FIG. VIII. f8:**
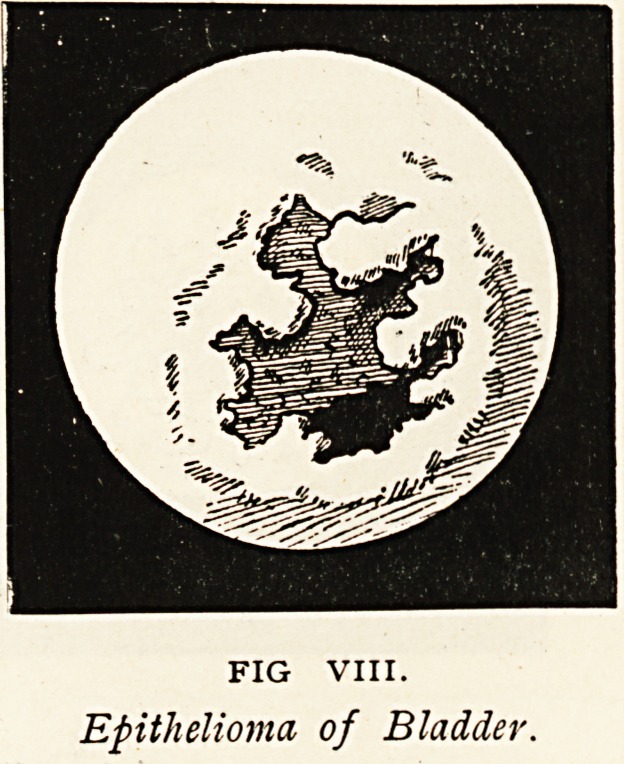


**FIG. IX. f9:**